# A dataset on trophic modes of aquatic protists

**DOI:** 10.3897/BDJ.8.e56648

**Published:** 2020-10-23

**Authors:** Lisa K. Schneider, Konstantinos Anestis, Joost Mansour, Anna A. Anschütz, Nathalie Gypens, Per J Hansen, Uwe John, Kerstin Klemm, Jon Lapeya Martin, Nikola Medic, Fabrice Not, Willem Stolte

**Affiliations:** 1 Deltares, Delft, Netherlands Deltares Delft Netherlands; 2 Université Libre de Bruxelles, Bruxelles, Belgium Université Libre de Bruxelles Bruxelles Belgium; 3 Alfred-Wegener-Institute Helmholtz Center for Polar and Marine Research, Bremerhaven, Germany Alfred-Wegener-Institute Helmholtz Center for Polar and Marine Research Bremerhaven Germany; 4 Sorbonne University, CNRS, Roscoff, France Sorbonne University, CNRS Roscoff France; 5 School of Earth and Ocean Sciences, Cardiff University, Cardiff, United Kingdom School of Earth and Ocean Sciences, Cardiff University Cardiff United Kingdom; 6 Marine Biological Laboratory, University of Copenhagen, Copenhagen, Denmark Marine Biological Laboratory, University of Copenhagen Copenhagen Denmark; 7 Helmholtz Institute for Functional Marine Biodiversity, Oldenburg, Germany Helmholtz Institute for Functional Marine Biodiversity Oldenburg Germany; 8 CNRS, Roscoff, France CNRS Roscoff France

**Keywords:** aquatic protists, phytoplankton, protozooplankton, mixoplankton, trophic mode, functional traits, functional biodiversity

## Abstract

**Background:**

An important functional trait of organisms is their trophic mode. It determines their position within food webs, as well as their function within an ecosystem. For the better part of the 20^th^ century, aquatic protist communities were thought to consist mainly of producers (phytoplankton) and consumers (protozooplankton). Phytoplankton cover their energy requirements through photosynthesis (phototrophy), while protozooplankton graze on prey and organic particles (phagotrophy). However, over the past decades, it was shown that another trophic group (mixoplankton) comprise a notable part of aquatic protist communities. Mixoplankton employ a third trophic mode by combining phototrophy and phagotrophy (mixotrophy). Due to the historical dichotomy, it is not straightforward to gain adequate and correct information on the trophic mode of aquatic protists. Long hours of literature research or expert knowledge are needed to correctly assign trophic modes. Additionally, aquatic protists also have a long history of undergoing taxonomic changes which make it difficult to compare past and present literature. While WoRMS, the World Register of Marine Species, keeps track of the taxonomic changes and assigns each species a unique AphiaID that can be linked to its various historic and present taxonomic hierarchy, there is currently no machine-readable database to query aquatic protists for their trophic modes.

**New information:**

This paper describes a dataset that was submitted to WoRMS and links aquatic protist taxa, with a focus on marine taxa, to their AphiaID and their trophic mode. The bulk of the data used for this dataset stems from (routine) monitoring stations in the North Sea and the Baltic Sea. The data were augmented and checked against state-of-the-art knowledge on mixoplankton taxa by consulting literature and experts. Thus, this dataset provides a first attempt to make the trophic mode of aquatic protists easily accessible in both a human- and machine-readable format.

## Introduction

Protists (i.e. unicellular eukaryotes) form the base of aquatic ecosystems by providing food for higher trophic levels. Even though protist communities are so important for the trophic functioning of aquatic ecosystems, the trophic diversity within those protist communities is not always clear. For the better part of the 20^th^ century, aquatic protist communities were divided into producers, the phytoplankton and grazers, the (proto)zooplankton (Flynn et al. 2013). Over the past decade, there has been an effort to reshape the traditional dichotomy of aquatic protist communities by taking mixotrophic protists into account (Mitra et al. 2016). Mixotrophic protists can function both as producers and grazers and, recently, the term mixoplankton has been suggested to describe this group (Flynn et al. 2019).

However, taking the correct trophic mode into account is still a challenge. Due to the historical bias, most aquatic protists are still by default categorised as either phytoplankton or protozooplankton. Intensive experimental work is required to determine mixotrophy in protists. While, in the past years, quite a few papers were published that contained lists of currently-proven marine mixoplankton (Faure et al. 2019, Leles et al. 2017, Leles et al. 2019), there is no database available which allows the trophic mode of aquatic protists to be queried. This makes it very time-consuming to take the trophic mode into account for large data-driven approaches on aquatic protist communities. A further complication is added through the frequent taxonomic changes within the protist community which make it difficult to compare literature references.

This dataset provides information on the trophic mode of aquatic protists and links them to the WoRMS database that keeps track of taxonomic name changes by using a unique species identifier, the AphiaID. By combining information on trophic modes with an already existing and widely-used database such as WoRMS, the authors hope to make data on trophic modes of aquatic protists more accessible in a machine-readable fashion. Thus, the dataset can help facilitate a better understanding of trophic dynamics and the functional role of protist groups within aquatic ecosystems. The trophic mode of the taxa included in this dataset can be accessed via the attributes of the WoRMS taxa search tool (see, for example, http://www.marinespecies.org/aphia.php?p=taxdetails&id=232772#attributes).

However, this dataset is only a start. The authors hope that, as more information on mixoplankton becomes available, this dataset will actively be expanded through community effort. New data can easily be submitted to WoRMS using the instructions available on https://www.marinespecies.org/contribute.php.

## General description

### Purpose

The purpose of this project was to establish a dataset on trophic modes of aquatic protists. As correct classification of trophic modes is especially important within the context of analysing routine monitoring data, the idea arose to make this work more accessible to the broad aquatic science and management community. This dataset was assembled in the scope of the H2020 Marie-Curie ITN "MixITiN".

## Project description

### Title

A dataset on trophic modes of aquatic protists

### Personnel

Lisa K. Schneider (management, data collection, literature research, tidy data implementation, data concatenation, manuscript preparation), Konstantinos Anestis (data collection, literature research, manuscript contribution), Joost Mansour (literature research, manuscript contribution), Anna A. Anschütz (literature research, manuscript revision), Nathalie Gypens (data collection, expert knowledge, manuscript revision), Per J. Hansen (data collection, expert knowledge, manuscript revision), Uwe John (data collection, expert knowledge, manuscript revision), Kerstin Klemm (data collection, manuscript revision), Jon Lapeya Martin (data collection, manuscript revision), Nikola Medic (literature research, manuscript revision), Fabrice Not (expert knowledge, manuscript revision), Willem Stolte (management, concept development, expert knowledge, manuscript preparation).

### Design description

To gather, analyse and disseminate the trophic mode of aquatic protists, a dataset was submitted to the World Register of Marine Species, WoRMS at http://www.marinespecies.org. WoRMS provides "an authoritative and comprehensive list of names of marine organisms, including information on synonymy" (WoRMS Editorial Board 2017) and this list of marine organisms can be augmented with metadata, such as traits, for example, trophic modes. Each organism is labelled with a unique AphiaID with which it is possible to keep track of taxonomic name changes (Vandepitte et al. 2015). This approach of keeping track of taxonomic name changes allows the database to be accessed and used in different ways, for example, by searching for single organisms or matching a list of taxa.

In this dataset, the trophic mode is defined by assigning one of the three different aquatic protist categories (*sensu* Flynn et al. (2019)): phytoplankton, protozooplankton or mixoplankton. Phytoplankton are defined as protists that obtain their nourishment via photo(auto)trophy and osmo(hetero)trophy. Irrespective of seasonality or environmental conditions, phytoplankton are always incapable of phago(hetero)trophy. Protozooplankton are defined as protists that obtain their nourishment via phago(hetero)trophy and osmo(hetero)trophy. Irrespective of seasonality or environmental conditions, protozooplankton are always incapable of photo(auto)trophy. Mixoplankton are defined as protists that obtain their nourishment by combining photo(auto)trophy, osmo(hetero)trophy and phago(hetero)trophy, so called mixotrophy.

Furthermore, for each mixoplankton, the type of mixotrophy is assigned as a trait. In the dataset, the type of mixotrophy is defined by assigning CM, GNCM, pSNCM or eSNCM to the mixoplankton, according to the types identified in Mitra et al. (2016). Constitutive Mixoplankton (CM) have the innate ability to perform photosynthesis, while Non-Constitutive Mixoplankton need to acquire chloroplasts from their prey. These Non-Constitutive Mixoplankton are divided into the General Non-Constitutive Mixoplankton (GNCM), the plastid Specialists Non-Constitutive Mixoplankton (pSNCM) and the endosymbiotic Specialist Non-Constitutive Mixoplankton (eSNCM). The GNCMs can use chloroplasts from multiple phototrophic prey, while the pSNCMs and eSNCMs only use chloroplasts from specific preys or endosymbionts.

### Funding

This project has received funding from the European Union's Horizon 2020 research and innovation programme under the Marie Skłodowska-Curie grant agreement No 766327 and EMODnet Biology (EC Service contract – EASME/EMFF/2016/1.3.1.2/Lot 5/SI2.750022).

## Sampling methods

### Study extent

This dataset focuses on the trophic modes of aquatic protists. It combines data from five different sources: routine monitoring (HELCOM 2019, Rijkswaterstaat 2019), scientific cruises (John 2020, Martin 2020), scientific papers (primary literature), review papers and a book chapter (secondary literature). Sampling for routine monitoring and on scientific cruises was performed using Niskin bottles, followed by inspection of the samples using light microscopy. In the case of the scientific cruises, metabarcoding was employed for further validation of the microscopic data. Suppl. material 1 lists all sources and their complete references.

### Sampling description

**Figure 1.**
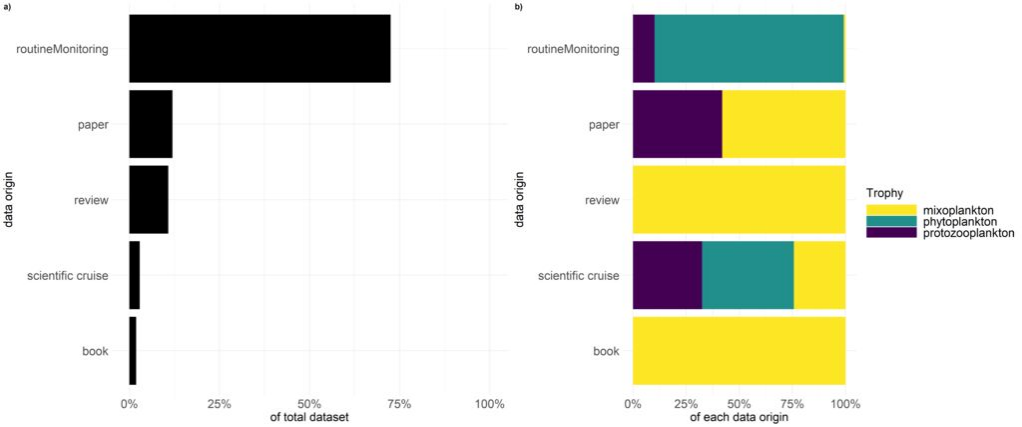
Depiction of a) percentage of the data origin to the complete dataset and b) percentage of trophic mode per data origin.

This dataset (Suppl. material 2) covers 1296 taxa. Fig. 1 shows that the bulk of the taxa stem from routine monitoring (72%) and are mainly labelled as phytoplankton (89%). Secondary literature (reviews and book chapter) contributes 10% and 1.9% of the total taxa, respectively, which are all labelled as mixoplankton. Primary literature (scientific papers) contributes 11% of the total taxa and are divided evenly between protozooplankton and mixoplankton. Recent scientific cruises in the North Sea contribute 3% of the total taxa and display the most even distribution of trophic modes.

**Figure 2.**
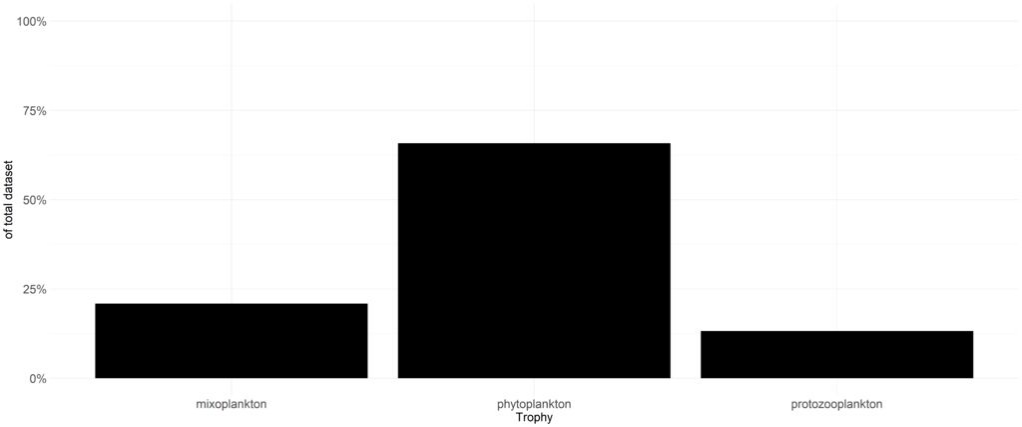
Contribution of trophic modes to total dataset.

In total, 21% of the taxa are classified as mixoplankton, 66% as phytoplankton and 13% as protozooplankton (Fig. 2). However, as 72% of the taxa originate from routine monitoring, the percentage of mixoplankton is most likely under-represented. Most routine monitoring undersample mixoplankton due to the employed sampling techniques (Flynn et al. 2019). An example is the routine monitoring data of the Dutch Southern North Sea in which ciliates, as well as nanoflagellates, are often not identified and counted. Both of these groups are known to contain mixoplankton (Haraguchi et al. 2018, Stoecker and Lavrentyev 2018). Furthermore, mixotrophy must be proven in phyto- and protozooplankton by observing either feeding or utilisation of chloroplasts from prey or symbionts. It must be assumed that taxa remain labelled according to the historical dichotomy until proven otherwise. This remains an issue also for this dataset, of which the user should be aware. It can only be remedied by continually updating the dataset as new mixoplankton taxa are empirically determined.

### Quality control

**Figure 3.**
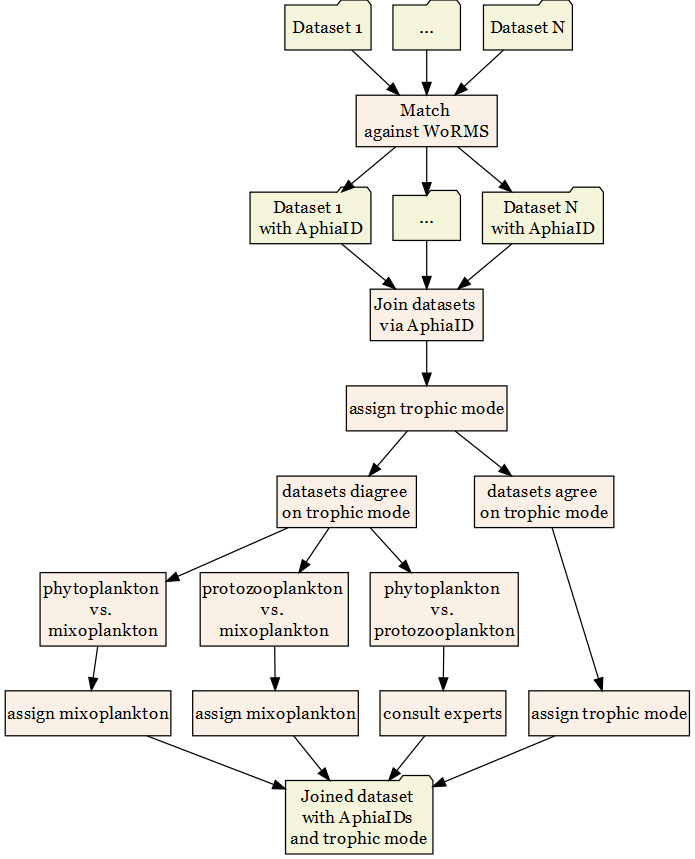
Workflow depiction beginning with single data sources and ending with the complete dataset.

In order to ensure consistent taxonomy over the various data sources, each data source was matched against the WoRMS taxonomic database using the WoRMS "Match taxa" tool. This ensured that each taxon was given the currently-accepted scientific name and referenced with an AphiaID. Data sources were tidied (Wickham 2014) and joined into one dataset. If the various sources disagreed with each other on the trophic mode of the taxon, two decision pathways were possible. Firstly, mixoplankton data sources were always given precedence over other sources. Secondly, if the data sources disagreed on non-mixoplankton, then expert knowledge and literature was used to assign the trophic mode of that organism. Lastly, the list was checked by expert witnesses to ensure correct trophic classifications. This described workflow is visualised in Fig. 3.

## Geographic coverage

### Description

As this consortium is based in the EU, data stemming from routine monitoring is biased towards European waters. Data derived from literature extends beyond the EU. We hope that this dataset will be built upon with data contributions from other scientists, to establish a more encompassing collaborative resource that will promote research on trophic modes. New data can easily be submitted to WoRMS using the instructions available on https://www.marinespecies.org/contribute.php.

## Taxonomic coverage

### Description

**Figure 4.**
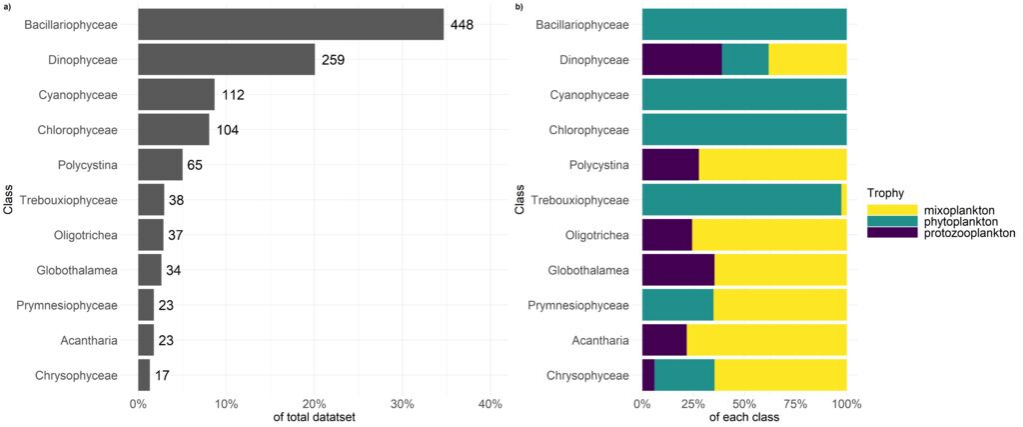
Depiction of a) percentage of each class to the complete dataset and b) percentage of trophic mode per class.

Fig. 4 visualises the contribution of each class to the total dataset (Fig. 4a), as well as the contribution of trophic modes across those classes (Fig. 4b). It should be noted that only those classes are displayed that make up 90% of the total dataset. The largest class, represented in the dataset, is Bacillariophyceae, followed by Dinophyceae and Cyanophyceae. In terms of trophy, the classes which contain the most phytoplankton are Bacillariophyceae, Cyanophyceae, Chlorophyceae and Trebouxiophyceae. The classes which contain the most mixoplankton are Dinophyceae, Polycystina, Oligotrichea, Globothalamea, Prymnesiophyceae, Acantharia and Cryptophyceae. Protozooplankton are represented in the classes Dinophyceae, Polycystina, Oligotrichea, Globothalamea and Acantharia, in which they contribute between 5% and 30%.

## Traits coverage

This dataset focuses on the trophic mode of aquatic protists. As mentioned, aquatic protists can be divided into phytoplankton, protozooplankton and mixoplankton. This next section will give more detail on these different functional groups and their impact on aquatic ecosystems.

Phytoplankton are defined as those protists that perform photosynthesis and are incapable of phagotrophy. The most prominent examples of phytoplankton groups are cyanobacteria, diatoms and green algae. Attributing to their need of light for photosynthesis, phytoplankton are found in the euphotic zone, where light is available. It is estimated that aquatic photosynthesis by phytoplankton totals about half of the total primary production on Earth (Falkowski 1994, Field 1998). Phytoplankton in marine ecosystems play an important role in major biogeochemical cycles. For example, cyanobacteria species are known for their capacity to fix atmospheric nitrogen (25-50% of global natural fixation), a unique feature for this phytoplankton group (Canfield et al. 2010). Furthermore, diatoms contribute considerably to the global carbon cycling as they are responsible for 30-40% of global primary productivity (Sarthou et al. 2005). The diatom cell wall is composed of silica and thereby diatoms are considered the world's largest contributors to the silica cycle (Tréguer and De La Rocha 2013). Moreover, sinking of phytoplankton contributes to the carbon export to the deep oceans (Falkowski 1994).

Protozooplankton are defined as those protists that gain their nutrition through capture and ingestion of prey (or organic particles). Protozooplankton do not have the capability for photosynthesis, nor other means of producing their own organic carbon. Examples of protozooplankton are heterotrophic ciliates, heterotrophic dinoflagellates and heterotrophic (nano)flagellates. The grazing of heterotrophic protists on phytoplankton plays an important role in controlling the growth and population of phytoplankton taxa. Heterotrophic protists are the connecting link for energy transfer towards higher trophic levels and, in some cases, can be responsible for the removal of the largest part of primary production (Calbet and Landry 2004, Lawerence and Menden-Deuer 2012). Apart from the consumption of phytoplankton, heterotrophic protists also ingest prokaryotes indicating their further involvement in planktic food web energy transfer (Cho et al. 2000, Pernice et al. 2014, Šimek et al. 2019).

Mixoplankton are defined as those protists that can combine phototrophy and phagotrophy (*sensu* Flynn et al. 2019). Mixoplankton are often associated with mature ecosystems (Mitra et al. 2014, Moorthi et al. 2017) and many harmful algal bloom species are known to be mixotrophic. Due to their ability to combine phototrophy and phagotrophy, they can simultaneously fulfil many of the functions (Selosse et al. 2016) described for both phytoplankton and protozooplankton. Mixoplankton can thus also contribute significantly to primary productivity and functionality of ecosystems (Ghyoot et al. 2017). The additional energy acquired by the consumption of prey can increase the gross growth efficiency of mixoplankton (Schoener and McManus 2012) and subsequently, have major effects on trophic transfer in the food web (Ward and Follows 2016). CMs (e.g. *Prymnesium parvum*, *Karlodinium veneficum*) that have the innate ability to perform photosynthesis, can express bacterivory (Unrein et al. 2007) or ingest other protists to supplement their nutritional needs (Stoecker et al. 2017). NCMs, such as the kleptoplastidic ciliate *Strombidium basimorphum*, can be voracious grazers, achieving grazing rates comparable with pure heterotrophic species (Maselli et al. 2020). Furthermore, NCMs also contribute significantly to primary production through either their ability to use prey chloroplasts after ingestion (Nielson et al. 2012) or others (eSNCMs, like many Acantharia and Foraminafera) through chloroplast containing endosymbionts (Caron et al. 1995).

## Usage rights

### Use license

Creative Commons Public Domain Waiver (CC-Zero)

## Data resources

### Data package title

Trophic modes of aquatic protists

### Number of data sets

2

### Data set 1.

#### Data set name

List of trophic citations

#### Number of columns

2

#### Description

List of trophic mode references giving the short citation form (used in the dataset) and the full reference.

**Data set 1.** 

Column label	Column description
source	Short citation of the reference source used in the dataset
Full citation	Full APA citation of the reference source

### Data set 2.

#### Data set name

Trophic modes of aquatic protists

#### Number of columns

13

#### Description

Dataset listing the trophic modes of aquatic protists (with reference and data source) along with their accepted scientific name, AphiaID and taxonomic hierarchy.

**Data set 2.** 

Column label	Column description
ScientificName	Accepted scientific name retrieved from WoRMS.
AphiaID	Accepted AphiaID (unique identifier) retrieved from WoRMS.
Trophy	Gives the trophic mode of taxa as either "phytoplankton", "protozooplankton" or "mixoplankon".
typeMX	Gives the type of mixotrophy as either "CM" (Consitutive Mixoplankton), "GNCM" (General Non-Constitutive Mixoplankton), "eSNCM" (endosymbiotic Specialist Non-Constitutive Mixoplankton) or "pSNCM" (plastid Specialist Non-Constitutive Mixoplankton). If the type of mixotrophy does not apply (because the organism is labelled as phytoplankton or protozooplankton), the type of mixotrophy is labelled with "NA".
source	Gives the reference for the assigned trophic modes. Refers to primary literature or secondary literature (book, review papers or published datasets from routine monitoring).
dataType	Denotes the origin of the data point. Can be either "book", "paper", "review", "routine monitoring" or "scientific cruise".
Kingdom	Kingdom of the taxa within the taxonomic hierarchy
Phylum	Phylum of the taxa within the taxonomic hierarchy
Class	Class of the taxa within the taxonomic hierarchy
Order	Order of the taxa within the taxonomic hierarchy
Family	Family of the taxa within the taxonomic hierarchy
Genus	Genus of the taxa within the taxonomic hierarchy
Species	Species of the taxa within the taxonomic hierarchy

## Additional information

A github repository is available which contains the code to match the species list against WoRMS, as well as to create the figures: https://github.com/lkschn/trophyProtists.

## Supplementary Material

8A351D38-A3D9-550E-BEA9-21A54A26E5FE 10.3897/BDJ.8.e56648.suppl1 Supplementary material 1 List of trophic citations Data type Short and full citations Brief description This list gives all trophic citations in their short form (used in the dataset), as well as their full reference. File: oo_444242.txt https://binary.pensoft.net/file/444242 Lisa K. Schneider, Konstantinos Anestis, Joost Mansour, Anna A. Anschütz, Nathalie Gypens, Per J. Hansen, Uwe John, Kerstin Klemm, Jon Lapeya Martin, Nikola Medic, Fabrice Not, Willem Stolte

7903AF66-04E2-5FF8-B057-9A1ABEF4C381 10.3897/BDJ.8.e56648.suppl2 Supplementary material 2 Dataset on trophic modes of aquatic protist Data type Trophic mode and taxonomy of aquatic protists Brief description This dataset lists the trophic modes of aquatic protists (with reference and data origin) along with their accepted scientific name, AphiaID and taxonomic hierarchy. File: oo_444243.csv https://binary.pensoft.net/file/444243 Lisa K. Schneider, Konstantinos Anestis, Joost Mansour, Anna A. Anschütz, Nathalie Gypens, Per J. Hansen, Uwe John, Kerstin Klemm, Jon Lapeya Martin, Nikola Medic, Fabrice Not, Willem Stolte
